# 73662 Racial Disparities in Septic Shock Mortality: Results from the OneFlorida Data Trust Cohort

**DOI:** 10.1017/cts.2021.700

**Published:** 2021-03-30

**Authors:** Lauren Page Black, Charlotte Hopson, Elizabeth DeVos, Michael Puskarich, Rosemarie Fernandez, Faheem Guirgis, Cynthia Garvan

**Affiliations:** 1 University of Florida College of Medicine – Jacksonville; 2 University of Florida College of Medicine - Jacksonville, Center for Data Solutions; 3 University of Florida College of Medicine – Jacksonville; 4 University of Minnesota; 5 University of Florida College of Medicine; 6 University of Florida College of Medicine – Jacksonville; 7 University of Florida College of Medicine

## Abstract

ABSTRACT IMPACT: Identifying racial disparities in septic shock mortality, a common and lethal condition, can inform future research and policy efforts aimed at understanding the drivers these disparities and addressing the underlying factors in order to reduce disparities and improve health. OBJECTIVES/GOALS: Septic shock is a major public health problem with significant mortality. Existing data indicate racial disparities in sepsis incidence, but evidence is limited on differences in septic shock outcomes. Our objective was to determine the association between race and septic shock mortality in a statewide cohort while controlling for clinical factors. METHODS/STUDY POPULATION: This was a retrospective analysis of septic shock patients in the One Florida Data Trust between 2012-18. Data was collected regarding age, sex, race, insurance status, and selected comorbid conditions [liver disease, hypertension, chronic obstructive pulmonary disease (COPD), congestive heart failure (CHF), end-stage renal disease (ESRD), and human immunodeficiency virus infection (HIV)]. To account for severity of illness, we assigned Sequential Organ Failure Assessment scores for components based on laboratory values (labSOFA), and collected data on mechanical ventilation use and initial lactate.

The primary outcome was 90-day mortality. The Least Absolute Shrinkage and Selection Operator (LASSO) method was used for variable selection for the multivariable regression model. RESULTS/ANTICIPATED RESULTS: There were 13,932 septic shock patients with a mean (SD) age of 61(16) years. Of these, 68% identified as white, 28% as black, 2.1% as Hispanic, and 2.0% as other races. 90-day mortality was 32% (n=4,437) and 59% required mechanical ventilation. Significant independent predictors of mortality in the regression model were age (OR 1.04; p<0.01), black race (1.72; p<0.01), lactate (1.10; p<0.01), mechanical ventilation (3.62; p<0.01), labSOFA (1.18; p<0.01), history of liver disease (1.75; p<0.01), hypertension (0.70; p<0.01), COPD (0.87; p<0.01), CHF (1.18; p<0.01), HIV (1.30; p=0.05), and the interaction between age and black race. Black patients had 1.72 times the odds of mortality compared to white patients. For every one-year decrease in age, black patients had a 1% increased odds of mortality (OR 1.01; p < 0.01). DISCUSSION/SIGNIFICANCE OF FINDINGS: Black patients have increased odds of dying from septic shock compared to white patients after controlling for age, selected comorbid conditions, and markers of illness severity. Future work is needed to move beyond demonstrating septic shock disparities and towards understanding the underlying factors.

